# Recovery of Chlorosilane Residual Liquid to Prepare Nano-Silica via the Reverse Micro-Emulsion Process

**DOI:** 10.3390/ma16216912

**Published:** 2023-10-27

**Authors:** Jixiang Cai, Youwen Li, Lianghuan Wei, Jiangpeng Xue, Ning Lin, Xianghao Zha, Guodong Fang

**Affiliations:** 1 Xinjiang Biomass Solid Waste Resources Technology and Engineering Center, College of Chemistry and Environmental Science, Kashi University, Kashi 844000, China; caijixiangedukashi@163.com (J.C.); liyouwen007@163.com (Y.L.); weilianghuanabc@126.com (L.W.); xuejiangpeng199119@163.com (J.X.); lintaoxp@163.com (N.L.); 2 Key Laboratory of Soil Environment and Pollution Remediation, Institute of Soil Science, Chinese Academy of Sciences, Nanjing 210008, China

**Keywords:** chlorosilane residual liquid, inverse micro-emulsions, nano-silica, sustainable development

## Abstract

In this paper, nano-silica particles were prepared from chlorosilane residue liquid using an inverse micro-emulsions system formed from octylphenyl polyoxyethylene ether (TX-100)/n-hexanol/cyclohexane/ammonia. The influence of different reaction conditions on the morphology, particle size, and dispersion of nano-silica particles was investigated via single-factor analysis. When the concentration of chlorosilane residue liquid (0.08 mol/L), hydrophile-lipophilic-balance (HLB) values (10.50), and the concentration of ammonia (0.58 mol/L) were under suitable conditions, the nano-silica particles had a more uniform morphology, smaller particle size, and better dispersion, while the size of the nano-silica particles gradually increased with the increase in the molar ratio of water to surfactant (ω). The prepared nano-silica was characterized through XRD, FT-IR, N_2_ adsorption/desorption experiments, and TG-DSC analysis. The results showed that the prepared nano-silica was amorphous mesoporous silica, and that the BET specific surface area was 850.5 m^2^/g. It also had good thermal stability. When the temperature exceeded 1140 °C, the nano-silica underwent a phase transition from an amorphous form to crystalline. This method not only promoted the sustainable development of the polysilicon industry, it also provided new ideas for the protection of the ecological environment, the preparation of environmental functional materials, and the recycling of resources and energy.

## 1. Introduction

With the continuous consumption of fossil fuels, the increasing risk of global climate degradation and the ongoing geopolitical turmoil, the energy crisis has also gradually intensified, which has led people to pay more attention to new energy and green, low-carbon transformation and development [1,2,3,4,5]. As a clean, green, and renewable energy, solar energy plays an important role in coping with climate change and the energy crisis [6]. However, polysilicon, as the main raw material for manufacturing solar cells, produces chlorosilane residue liquid composed of silicon tetrachloride (SiCl_4_), trichlorosilane (SiHCl_3_), and dichlorosilane (SiH_2_Cl_2_) during its production process, which has a strong corrosiveness and toxicity [7,8,9]. Therefore, the adoption of efficient and applicable methods for resource treatment and the utilization of chlorosilane residue liquid is not only beneficial for environmental protection and resource recycling, but also of great significance for the sustainable development of the polysilicon industry.

However, nano-silica has the characteristics of a large specific surface area, low toxicity, good optical transparency, excellent biological compatibility, and easy functional modification. It not only shows the small size effect, macro quantum effect and surface effect of general nano-materials, it also has unique physical and chemical properties such as photoelectric properties, a high magnetoresistance phenomenon, and nonlinear resistance [10,11]. It was widely used in the fields of material adsorption and separation, electronic packaging materials, nano-biomedicine, antimicrobial materials, catalysis, and drug carriers [12,13], which made use of the chlorosilane residue liquid to prepare nano-silica showing a high value and unique advantages in terms of resource utilization and environmental protection.

At present, the main methods for the preparation of nano-silica include vapor phase hydrolysis [14], chemical precipitation [15,16], solgel [17,18], and inverse micro-emulsions methods [19]. Among them, inverse micro-emulsions were usually a uniform and stable dispersion system composed of surfactant, co-surfactant, oil phase, and water phase, all of which had a translucent to transparent appearance [20]. In the reaction process, the reactants were compatibilized into the water nucleation reactor formed via the inverse micro-emulsions, so that the whole process of particle nucleation and growth was bound by the water nucleation reactor. In addition, there was a large amount of oil phase and interfacial film composed of surfactant and co-surfactant outside the water nucleation reactor, which hindered the further agglomeration of the product nanoparticles, and thus the prepared silica particles were smaller in size, more uniformly distributed, and better dispersed [21]. At the same time, the shape and size of the water nucleation reactor was controlled by adjusting the composition of the inverse micro-emulsions, which effectively regulated the particle size, morphology, and dispersion of the generated nanoparticles, making the prepared nanoparticles easy to modify and control and thus, greatly improving their utilization value [22].

Therefore, in this paper, nano-silica was prepared through treating chlorosilane residue liquid with inverse micro-emulsions composed of TX-100 as the surfactant. The effects of different influencing factors on the particle size, morphology, and dispersion of the nano-silica were investigated through adjusting the reaction conditions [23]. At the same time, the properties of nano-silica were analyzed through combining various characterization methods. Through realizing the resource utilization of the chlorosilane residue liquid and promoting the sustainable and green development of polysilicon production industry, it provided a new way for the protection of the ecological environment and the preparation of environmental functional materials through the recycling of resources and energy.

## 2. Experimental Section

### 2.1. Materials

The chlorosilane residual liquid with the main composition of SiCl_4_ (80.67%), SiHCl_3_ (9.86%), and HCl (9.47%) was supplied by Yunnan Metallurgical Yunxin Silicon Materials Co., Ltd., Kunming, China. Biochemical reagent grade octylphenyl polyoxyethylene ether (TX-100) was purchased from Aladdin Reagent (Shanghai) Co., Ltd., Shanghai, China. Analytical grade cyclohexane, n-hexanol, ammonia, acetone, and absolute ethanol were all purchased from Tianjin Chemical Co., Ltd., Tianjin, China. Deionized water was used for the experiments.

### 2.2. Preparation of Silica

#### 2.2.1. Preparation Method of Silica

The surfactant (TX-100), co-surfactant (n-hexanol), and oil phase (cyclohexane) were mixed in a certain proportion and then ammonia was added dropwise to the mixture in a thermostatically heated magnetic stirring water bath. After thorough mixing and emulsification, inverse micro-emulsions containing ammonia (denoted as phase A) were formed. It was then left to stand for 1 h. Using the same method and keeping other conditions unchanged, the inverse micro-emulsions containing chlorosilane residue liquid (recorded as phase B) were prepared through replacing the ammonia in phase A with chlorosilane residue liquid. They were also left to stand for 1 h.

In a three-necked flask reactor under atmospheric pressure and without the use of an inert atmosphere, the inverse micro-emulsions (B) containing chlorosilane residue liquid were dropped into the inverse micro-emulsions (A) containing ammonia, and stirred in a thermostatically heated magnetic stirring water bath at a medium speed for 2 h at 30 °C to make it fully react. The system was then transparent or white translucent and was left to stand at 30 °C for 24 h. The reaction system with acetone added for demulsification was centrifuged at 10,000 r/min with a high-speed centrifuge for 10 min to obtain the silica. In order to remove the residual surfactant of silica, we used anhydrous ethanol to ultrasonic clean it at least 3 times. Finally, the prepared sample was dried in a constant temperature drying oven at 80 °C for 6 h to obtain white silica particles. The reactions were shown in Equations (1)–(3).

SiH_2_Cl_2_ + 2H_2_O → SiO_2_ + HCl + 2H_2_ (1)

SiHCl_3_ + 2H_2_O → SiO_2_ + 3HCl + H_2_ (2)

SiCl_4_ + 2H_2_O → SiO_2_ + 4HCl (3)

#### 2.2.2. Effect of Reaction Conditions on Silica

According to the above silica preparation method, the effects on the particle size, morphology, and dispersion of the prepared nano-silica were studied via varying the influencing factors such as the concentration of chlorosilane residual liquid (0.04 mol/L, 0.08 mol/L, 0.12 mol/L, 0.16 mol/L, 0.20 mol/L), the value of HLB (13.50, 11.63, 10.50, 9.75, 9.21), molar ratio of water to surfactant (ω) (9, 12, 16, 20, 32), and the concentration of ammonia (0.15 mol/L, 0.30 mol/L, 0.44 mol/L, 0.58 mol/L, 0.65 mol/L).

#### 2.2.3. Particle Characterization

Utilizing field emission scanning electron microscopy (FE-SEM, FEL Nova NANOSEM-450, Thermo Fisher, Waltham, MA, USA) combined with Image J analysis software (Image J. v2X), the distribution, structure and, diameter of silica particles were characterized [24]. The crystal structure of silica was examined through X-ray diffraction (XRD, Empyrean, Amsterdam, PANalytical B.V., Alemlo, The Netherlands) in a 2θ range of 5 to 80°. The molecular structure of silica was analyzed via Fourier transform infrared (FT-IR) spectroscopy (VERTEX70, Bruker Corporation, Mannheim, Germany) and the KBr method (signal-to-noise ratio of 45,000:1, resolution of 0.4 cm^−1^, and wave number range of 400–4000 cm^−1^). Nitrogen adsorption and desorption experiments and Brunauer Emmett Teller (BET) specific surface area measurements were used to analyze the specific surface area and pore size distribution of silica (the samples were vacuum degassed at 90 °C for 1 h before the nitrogen adsorption and degassing experiments and then vacuum degassed at 160 °C for 2 h. The nitrogen adsorption and degassing experiments were carried out at a temperature of 77 K). In addition, thermogravimetric analysis was also used to evaluate the thermal stability of the silica (TG, STA 449 F3 Germany, temperature range of RT-1400 °C, heating rate of 0.001–50 K/min, and nitrogen as a protective gas).

## 3. Results and Discussion

### 3.1. Effect of Different Reaction Components on Silica

#### 3.1.1. Effect of Chlorosilane Residue Liquid Concentration on the Particle Size and Morphology of Nano-Silica

**Figure 1:**
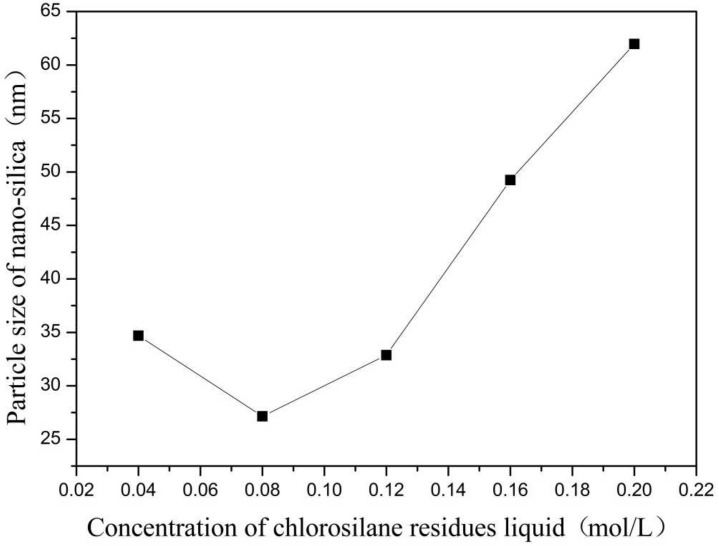
Effect of concentration of chlorosilane residues liquid on particle size of nano-silica. ■ represents the particle size of nano-silica.

**Figure 2:**
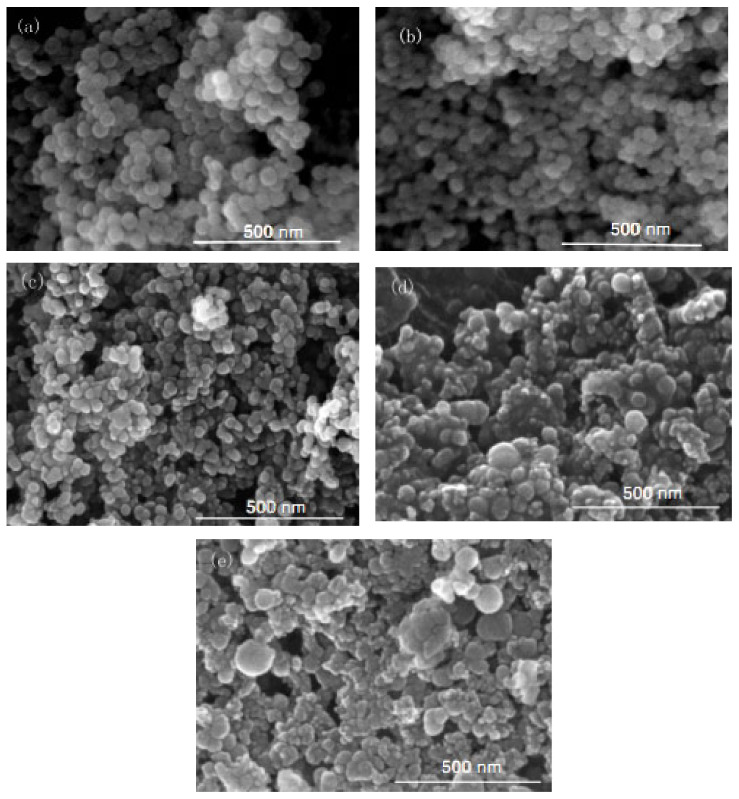
FE-SEM of nano-silica under different concentrations of chlorosilane residues liquid: (**a**) 0.04 mol/L, (**b**) 0.08 mol/L, (**c**) 0.12 mol/L, (**d**) 0.16 mol/L, (**e**) 0.2 mol/L.

In this group of experiments, the inverse micro-emulsions (A) containing ammonia were kept constant, the concentration of chlorosilane residue liquid in inverse micro-emulsions (B) was varied within the range of inverse micro-emulsions phase diagram, and other conditions were kept unchanged. The specific composition of the inverse micro-emulsions (B) is shown in Table 1. The inverse micro-emulsions (A) were mixed with the inverse micro-emulsions (B) shown in Table 1, respectively, and then magnetically stirred for 2 h and aged at 30 °C for 24 h. The influence of the concentration of its chlorosilane residue liquid on the particle size of the nano-silica is shown in Figure 1, and the SEM photos of the reaction products are shown in Figure 2.

It can be seen in Figure 1 that the particle size of the reaction product SiO_2_ particles showed an overall increasing trend with the increase in the concentration of chlorosilane residue liquid, with an average particle size of 34.69 nm, 27.16 nm, 32.88 nm, 49.25 nm, and 61.96 nm, respectively. In addition, the agglomeration of SiO_2_ particles became more and more serious (Figure 2).

The main reason for this phenomenon was that when the concentration of chlorosilane residue liquid was very low, in the inverse micro-emulsions system containing chlorosilane residue liquid, there were few chlorosilane residue liquid molecules in each inverse micro-emulsions micelle. When they reacted with the inverse micro-emulsions system containing ammonia, they generated fewer SiO_2_ particles in each inverse micro-emulsion in the water nuclear reactor. Since the diameter of each water nuclear reactor was certain, the generated SiO_2_ particles had enough space in the water nuclear reactor to enable them to grow continuously, which led to the increase in the particle size of SiO_2_ particles. At the same time, because there were a lot of oil phases around the water nuclear reactor, they played a certain role in protecting the generated SiO_2_ particles, preventing the SiO_2_ particles in different water nuclear reactors from colliding and agglomerating. With the increasing concentration of chlorosilane residual liquid, there were more and more chlorosilane residual liquid molecules in the inverse micro-emulsions in the water nuclear reactor. The number of SiO_2_ particles generated also increased with these particles competing to grow in the limited space of the water nuclear reactor. The growth space of each SiO_2_ particle became smaller and its particle size also became smaller. At the same time, in this process, the interfacial film composed of surfactant and co-surfactant separated the SiO_2_ particles generated in the reaction so that they did not contact and collide, thus preventing them from agglomerating. When the concentration of chlorosilane residue liquid continued to increase, the SiO_2_ particles generated in the inverse micro-emulsions in the water nuclear reactor gradually exceeded the maximum amount it could accommodate. At this time, the SiO_2_ particles began to break through the barrier of the interfacial film and continued to gather in the water nuclear reactor, until they completely broke the reactor bondage. At that moment, the whole reaction lost control of the inverse micro-emulsion system, so that the generated SiO_2_ particles in a disorderly state and contact, collided and grew with other particles. In addition, due to the loss of the protection of the water nuclear reactor, SiO_2_ particles also gradually agglomerated.

#### 3.1.2. Influence of Different HLB Values on the Particle Size and Morphology of Nano-Silica

**Figure 3:**
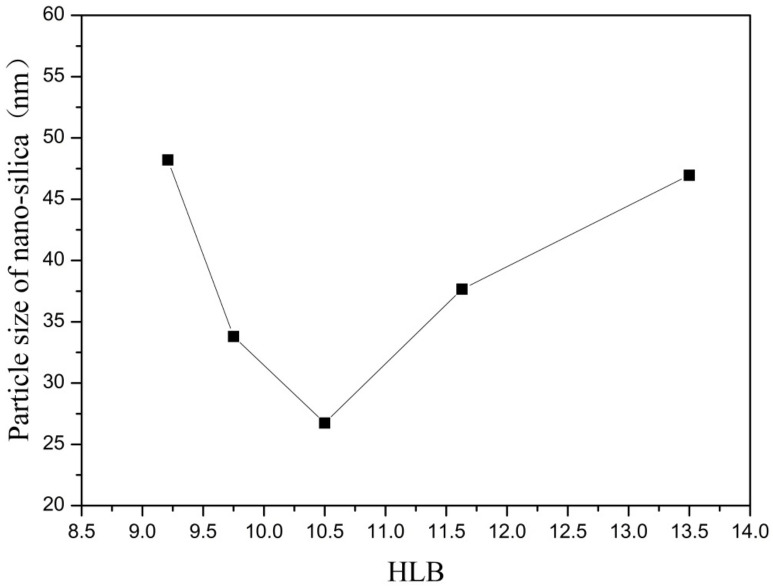
Effect of HLB on particle size of nano-silica. ■ represents the particle size of nano-silica.

**Figure 4:**
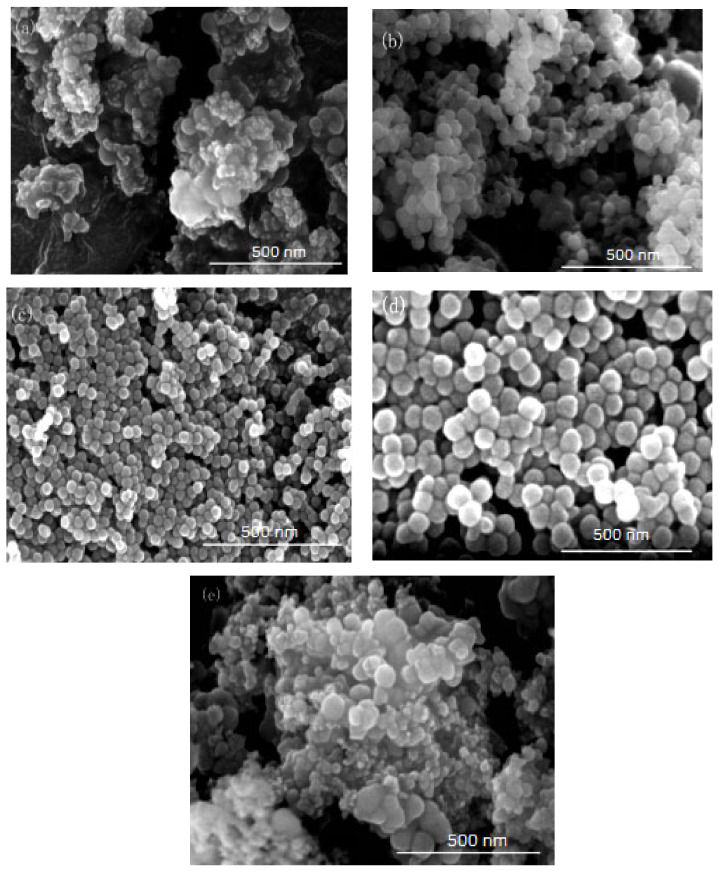
FE-SEM of nano-silica under different HLB values: (**a**) 13.50, (**b**) 11.63, (**c**) 10.50, (**d**) 9.75, (**e**) 9.21.

In this group of experiments, the amount of co-surfactant (n-hexanol) in the inverse micro-emulsions reaction system was changed, thus changing the mass ratio of TX-100/n-hexanol, so that the reaction system showed different HLB values. The other conditions remained unchanged, with the specific composition of the reaction system shown in Table 2. The inverse micro-emulsions (A) containing ammonia and the inverse micro-emulsions (B) containing chlorosilane residue liquid were mixed according to the ratios shown in Table 2, and then magnetically stirred for 2 h and aged at 30 °C for 24 h. The effect of the HLB values on the particle size of nano-silica under different co-surfactant masses is shown in Figure 3 and the SEM photographs of the reaction products are shown in Figure 4.

It can be seen from Figure 3 and Figure 4 that when there was no co-surfactant n-hexanol in the inverse micro-emulsions system or the amount of n-hexanol was low, the surface tension of the reaction system was larger, which made the inverse micro-emulsions system more difficult to form. Even when the inverse micro-emulsions were formed, the interfacial film strength of the surfactant was low, which made the rate of material exchange between the reactant particles too fast, thus making the size of the prepared SiO_2_ particles too large and with poor dispersion; the agglomeration phenomenon appeared when serious. With the increase in the co-surfactant dosage, when the amount of n-hexanol was suitable, the tension of the oil-water interface in the system was further reduced and the fluidity and flexibility of the interface were enhanced. This reduced the energy required in the process of forming the inverse micro-emulsions and made it easier to form inverse micro-emulsions. At the same time, the interfacial film strength of the inverse micro-emulsions system had also been enhanced to a certain extent, so that the whole reaction system became more stable, leading to a smaller particle size and uniform distribution of reaction products. When the amount of n-hexanol continued to increase, the carbon chain length of the alcohol was shorter than that of the surfactant, resulting in the gap of the interfacial film becoming larger. This reduced the strength of the interfacial film and the stability of the inverse micro-emulsions system became worse, resulting in the agglomeration phenomenon and the particle size of the reaction product SiO_2_ becoming larger.

On the other hand, changing the mass ratio of surfactant (TX-100) to co-surfactant (n-hexanol) affected the HLB of the whole inverse micro-emulsions system, thus affecting the amount of solubilized water phase in the inverse micro-emulsions system and its stability. When the amount of co-surfactant (n-hexanol) started to decrease, the HLB value of the inverse micro-emulsions system started to increase and the hydrophilicity of the interfacial system composed of surfactant and co-surfactant gradually increased. The lipophilicity, however, became weaker so that the system solubilized more water phase. With the decreased amount of n-hexanol, the hydrophilicity and lipophilicity of the inverse micro-emulsions system gradually reached a balance and the amount of solubilized water phase in the system reached its maximum. When the amount of n-hexanol continued to increase, the amount of water phase in the inverse micro-emulsions system was too large, which destroyed the hydrophilic lipophilic balance of the system and made the stability of the whole system worse. Therefore, when the HLB value of the inverse micro-emulsions system was 10.50, the stability of the system was at its best; the prepared reaction product had a smaller particle size and better dispersion.

#### 3.1.3. Effect of Different Molar Ratios of Water and Surfactant (ω) on the Particle Size and Morphology of Nano-Silica

**Figure 5:**
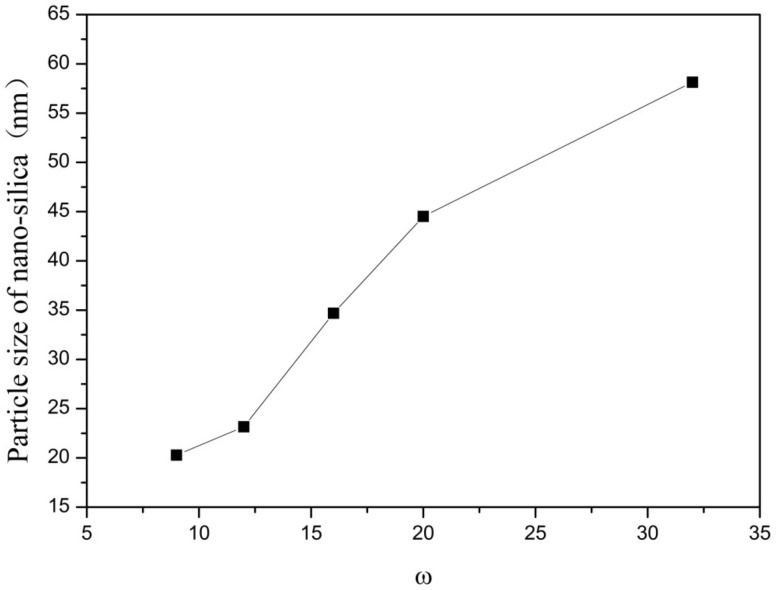
Effect of ω on particle size of nano-silica. ■ represents the particle size of nano-silica.

**Figure 6:**
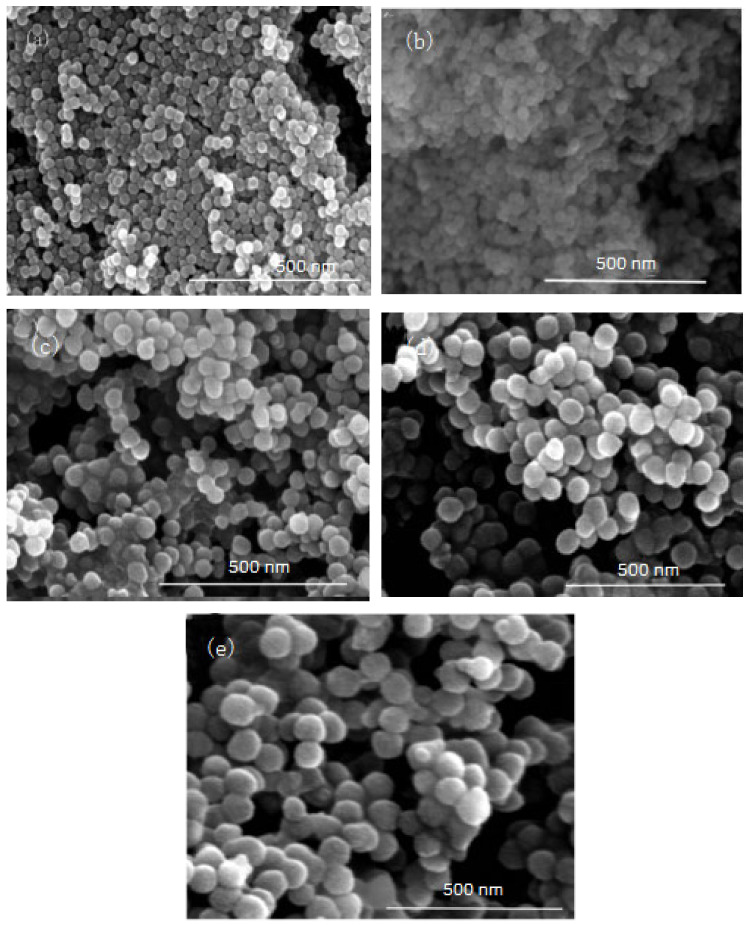
FE-SEM image of nano-silica under different ω: (**a**) 9, (**b**) 12, (**c**) 16, (**d**) 20, (**e**) 32.

In this group of experiments, the mass of ammonia in the inverse micro-emulsions system (A) containing ammonia water was ensured to be constant. Through adding different masses of distilled water, the molar ratio of water to surfactant (ω) was changed, with other conditions remaining unchanged. The specific composition is shown in Table 3. The inverse micro-emulsions (B) containing chlorosilane residue liquid were mixed with the inverse micro-emulsions (A) shown in Table 3, respectively, and then magnetically stirred for 2 h and aged at 30 °C for 24 h. The effect of the molar ratio of water to surfactant (ω) on the particle size of the nano-silica is shown in Figure 5 and the SEM photographs of the reaction products are shown in Figure 6.

It can be seen from Figure 5 and Figure 6 that with the increase of ω in the inverse micro-emulsions system, the particle size of the reaction product SiO_2_ gradually became larger and its dispersion gradually became worse, as well as the agglomeration phenomenon appearing. This was because in the inverse micro-emulsions system, the molar ratio of water to surfactant (ω) directly affected the size of the water nucleus of the inverse micro-emulsions and the size of the water nucleus of the m inverse micro-emulsions affected the size of the formed water nucleus reactor, which then affected the size and morphology of the generated SiO_2_ particles. The change of ω changed the interfacial film strength of the inverse micro-emulsions system, thus affecting the ability and rate of material exchange in the water nucleation reactor. Restricting the contact, collision, and growth between the two reactants resulted in a change in the size and morphology of the reaction products. Generally, in the inverse micro-emulsions system, when the ω value was very small, water molecules and hydrophilic groups of surfactants combined to form bound water. At this time, during the reaction, there were fewer SiO_2_ particles generated in the water nuclear reactor and their particle size was also smaller and almost non-agglomerated. With the increase in solubilized water in the inverse micro-emulsions, the ω also started to increase and the water molecules in the inverse micro-emulsions system mainly existed as irreducible water and free water. The more water that was solubilized to the inverse micro-emulsions system, the larger the proportion of free water. The size of the water nucleus reactor also became larger, leading to a reduction in the strength of the W/O interfacial film composed of surfactant and co-surfactant, as well as the stability of the water nuclear reactor. At the same time, the rate of material exchange in the water nucleus reactor was accelerated, which eventually made the particle size of the reaction product SiO_2_ larger and increased the possibility of agglomeration.

#### 3.1.4. Influence of Ammonia Concentration on the Particle Size and Morphology of Nano-Silica

**Figure 7:**
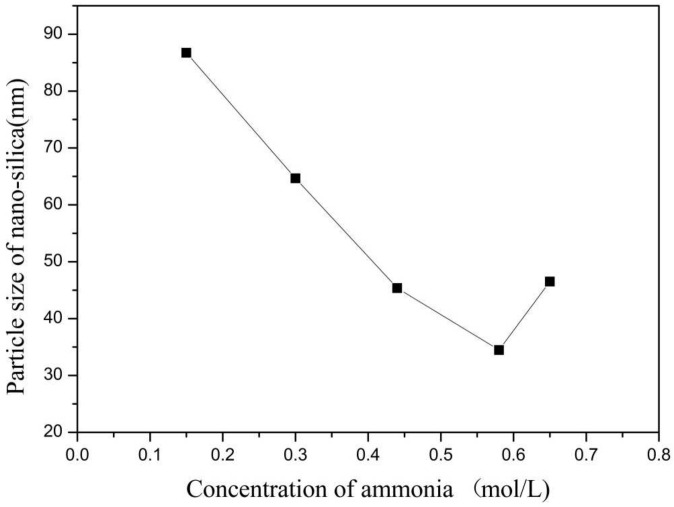
Effect of concentration of ammonia on particle size of nano-silica. ■ represents the particle size of nano-silica.

**Figure 8:**
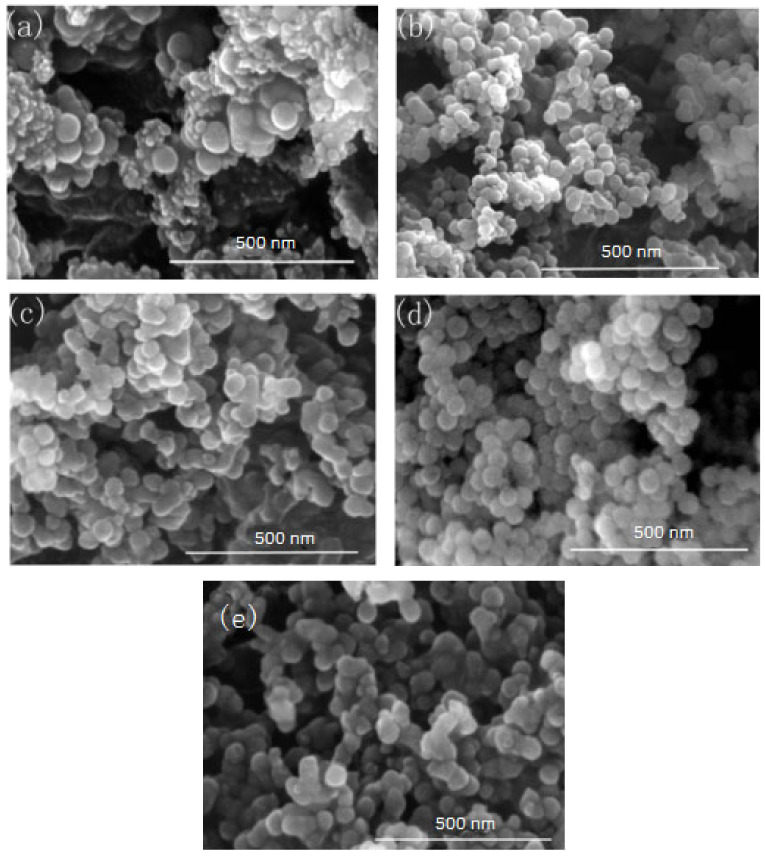
FE-SEM of nano-silica under different ammonia concentration: (**a**) 0.15 mol/L, (**b**) 0.3 mol/L, (**c**) 0.44 mol/L, (**d**) 0.58 mol/L, (**e**) 0.65 mol/L.

In this group of experiments, the concentration of ammonia in inverse micro-emulsions A was changed. Distilled water was added to the system to keep the molar ratio of water to surfactant (ω) constant with other conditions unchanged. The specific composition is shown in Table 4. The inverse micro-emulsions (B) containing chlorosilane residue liquid were mixed with the inverse micro-emulsions (A) shown in Table 4, respectively, and then magnetically stirred for 2 h and aged at 30 °C for 24 h. The effect of the concentration of ammonia on the particle size of nano-silica is shown in Figure 7 and the SEM photographs of the reaction products are shown in Figure 8.

It can be seen from Figure 7 and Figure 8 that when the ammonia concentration in the inverse micro-emulsions system was small, the reaction product SiO_2_ particles had a large particle size, poor dispersion, and an appearance of the agglomeration phenomenon. This was because the chlorosilane residue liquid itself contained a certain amount of HCL (partially generated during the hydrolysis reaction) so that the entire inverse micro-emulsions reaction system was under acidic conditions, while the main components SiCl_4_ and SiHCl_3_ in the chlorosilane residue liquid underwent hydrolysis reaction under acidic conditions to generate orthosilicic acid (Si(OH)_4_) and SiHCl_3._ This underwent hydroxylation polymerization under acidic conditions. When the concentration of orthosilicic acid was too low, it was easy to form sol and when the concentration of orthosilicic acid was high, it was easy to form gel so that the size of SiO_2_ particles generated via the reaction became larger and the dispersion became worse due to agglomeration. With the increase in ammonia concentration, the pH of the whole inverse micro-emulsions system increased continuously until the system became alkaline. Under alkaline conditions, the inverse micro-emulsions system contained a large amount of OH^-^, and when chlorosilane residue liquid hydrolyzed with it, SiCl_4_ and SiHCl_3_ no longer generated sol or gel, yet directly generated SiO_2_ particles. The by-products hydrochloric acid and ammonia generated ammonium chloride. In addition, alkaline conditions were conducive to dehydration and polycondensation of silicic acid molecules. These all led to a smaller particle size, more uniform dispersion, less agglomeration, or almost no agglomeration of SiO_2_ particles generated via the reaction. When the concentration of ammonia was further increased, it destroyed the hydrogen bond of the aqueous solution in the inverse micro-emulsions system, thus weakening the hydrophilicity of the surfactant and destroying the stability of the inverse micro-emulsions system. This led to an increase in the rate of material exchange in the inverse micro-emulsions in the water core reactor, making the generated SiO_2_ particles gather. They then continued to grow, thus making the final generated SiO_2_ particles larger in size and worse in dispersion and agglomeration. Therefore, when the mass ratio of ammonia to cyclohexane was 1:11.25 and ω was 20, the nano-silica prepared via the method had a smaller particle size and a more uniform dispersion.

### 3.2. Characterization of Nano-Silica Performance

When the concentration of chlorosilane residue liquid was 0.08 mol/L, the value of HLB was 10.50, the molar ratio of water to surfactant was 20 and the concentration of ammonia was 0.58 mol/L, the prepared nano-silica particles had a more uniform morphology, smaller particle size, and better dispersibility. In order to better explore the physical and chemical properties of the prepared nano-silica particles, it was necessary to use various methods to characterize them.

#### 3.2.1. Phase Analysis of Nano-Silica

**Figure 9:**
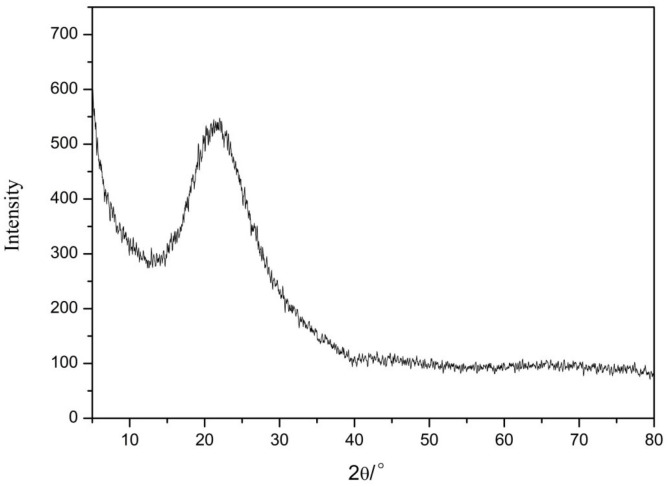
The X-ray diffraction pattern of the nano-silica.

The XRD results of nano-silica are presented in Figure 9. We found that there were no crystal diffraction peaks or other impurity peaks, while only a relatively broad dispersion peak appeared with 2θ of 21° to 24° according to the XRD card (JCPDS NO. 2920085). These results indicated that this peak was a characteristic peak of silica, thus proving that the prepared nano-silica was an amorphous silica [24].

#### 3.2.2. FT-IR Analysis of Nano-Silica

**Figure 10:**
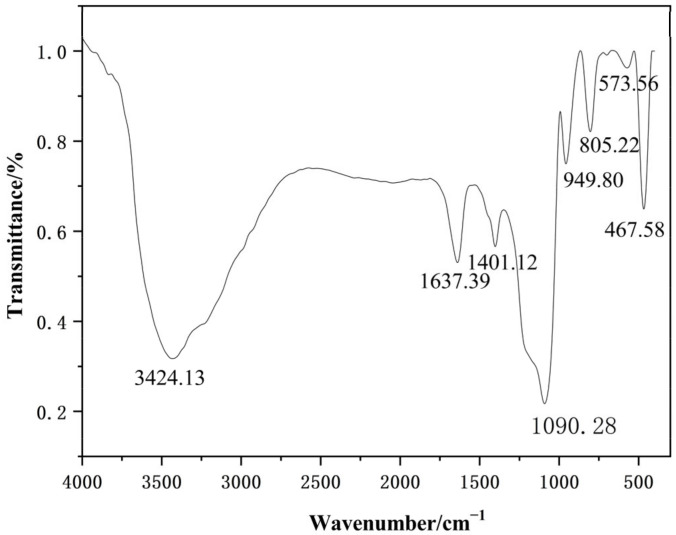
The Fourier transform infrared spectroscopy of the nano-silica.

The FT-IR analysis of the nano-silica samples was performed to determine the group structure of the nano-silica particle, the results of which are shown in Figure 10. The absorption bands at 467.58 cm^−1^, 805.22 cm^−1^, and 1090.28 cm^−1^ provided crucial information about the molecular composition and structure of nano-silica, with these peaks corresponding to the bending vibrational peaks, symmetrical vibrational peaks, and antisymmetrical vibrational peaks of Si-O-Si bonds, respectively, which were distinctive identifiers of nano-silica [25,26]. Furthermore, vibration peaks indicating a silicon-oxygen coupling bond, Si-OH bond, and H-O-H bond were found at 573.56 cm^−1^, 949.80 cm^−1^, and 1637.39 cm^−1^, respectively [27,28]. In addition, the vibrational peaks representing the isolated silanols or terminal silanols and C-O bond were also displayed at 3424.13 cm^−1^ and 1401.12 cm^−1^, respectively [29,30].

#### 3.2.3. Pore Structure Analysis of Nano-Silica

**Figure 11:**
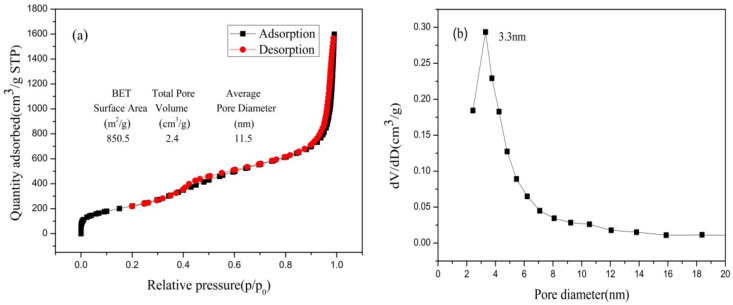
N_2_ adsorption-desorption isotherms and pore size distribution of nano-silica samples: (**a**) BET curve; (**b**) pore diameter.

Figure 11 presents the isotherm for nitrogen adsorption-desorption and the distribution of pore sizes of nano-silica. The consistency of nitrogen adsorption-desorption isotherms with the International Union of Pure and Applied Chemistry (IUPAC) classified as type IV and H3 type hysteresis loop were observed in the nano-silica samples, which implied that the nano-silica prepared via the present method had a mesoporous structure. The pore size distribution of the nano-silica particles were of mesoporous size, and their specific surface area which was calculated via the BET method, was 850.5 m^2^/g. Compared with the preparation of nano-silica using other silicon sources and methods, the nano-silica prepared in this study had a smaller particle size and a larger specific surface area (see Table 5), which indicated that the nano-silica prepared in this study had a wider range of applications and a higher application value.

#### 3.2.4. Thermal Stability Analysis of Nano-Silica

**Figure 12:**
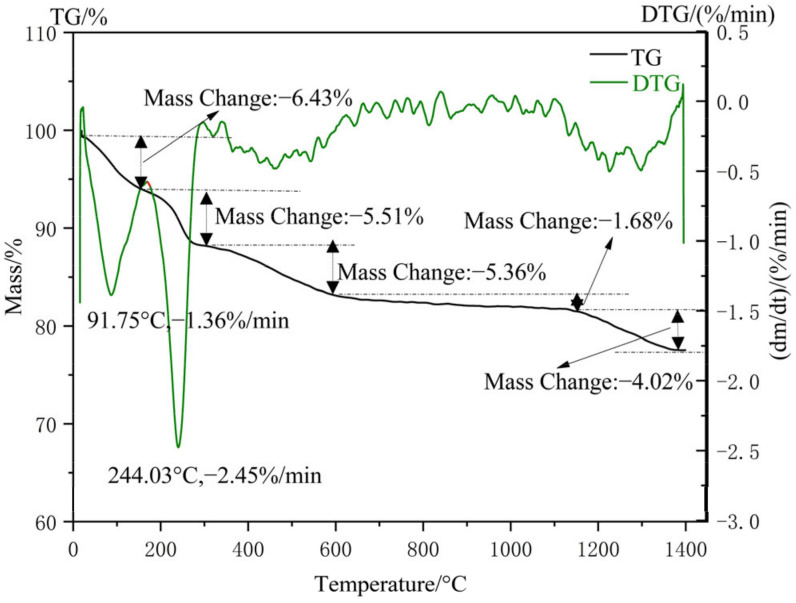
TG-DSC profiles of nano-silica samples.

The TG-DSC analysis result of nano-silica from the thermogravimetric analyzer is shown in Figure 12. In the whole process of thermal stability analysis, there were mainly five significant weight losses. During the temperature range from 0 °C to 158 °C, the free water and adsorbed water of nano-silica particles was removed due to the rise in temperature (weight loss of 6.43%). The hydrogen-bonded water of silica was removed at a temperature of 158 °C to 310 °C, resulting in a 5.51% weight loss of silica. In the temperature range of 310 to 600 °C, due to the conversion of Si-OH bonds into Si-O-Si bonds in silica and the volatilization of some organic compounds, there was a 5.36% weight loss of silica. The main reason for the 1.68% weight loss of nano-silica was that the connected and adjacent hydroxyl groups were further dehydrated and condensed within the range of 600 °C to 1140 °C. Nano-silica transformed from an amorphous state to a crystalline structure at temperatures exceeding 1140 °C and caused a weight loss of 4.02%. In addition, the evaporation of ethanol and bound water were also evidenced by the endothermic peaks at 91.75 °C and 244.03 °C in the TDS curves, respectively.

## 4. Conclusions

In this paper, nano-silica was prepared from chlorosilane residual liquid in a reverse micro-emulsion system formed from TX100, n-hexanol, cyclohexane, and ammonia. The effects of the concentration of chlorosilane residue liquid, the value of HLB, the molar ratio of water to surfactant (ω), and the concentration of ammonia on the morphology, particle size, and dispersion of nano-silica particles were investigated via single-factor analysis. It was found that the morphology, particle size, and dispersion of nano-silica particles can be effectively controlled through adjusting and changing the reaction conditions. When the concentration of chlorosilane residue liquid (0.08 mol/L), the value of HLB (10.50), and the concentration of ammonia (0.58 mol/L) were under suitable conditions, the prepared nano-silica particles had a more uniform morphology, smaller particle size, and better dispersion. However, the particle size of nano-silica particles gradually increased with the increase in the molar ratio of water to surfactant (ω). In addition, various characterizations showed that nano-silica had impressive properties, such as an amorphous structure, a specific surface area of 850.5 m^2^/g, a pore volume of 2.4 cm^3^/g, and a pore size distribution of 11.5 nm. During the continuous heating, water and hydroxyl groups underwent removal which led to a significant reduction in the weight of the nano-silica. When the temperature exceeded 1140 °C, the phase transition of nano-silica occurred, changing from an amorphous state into crystalline. The utilization of the reverse micro-emulsion technique for treating the liquid residue of chlorosilane and synthesized nano-silica not only promoted the sustainability development of polysilicon industry, but also provided a new way to protect the ecological environment, the preparation of environmental functional materials, and the recycling of resources and energy.

## Figures and Tables

**Table 1:** The component of inverse micro-emulsions (B) at different concentrations of chlorosilane residue liquid.

No.	TX-100/n-Hexanol (Mass Ratio)	Ammonia/Cyclohexane (Mass Ratio)	Concentration of Chlorosilane Residue Liquid (mol/L)
a	3:2	1:10	0.04
b	3:2	1:10	0.08
c	3:2	1:10	0.12
d	3:2	1:10	0.16
e	3:2	1:10	0.20

**Table 2:** The component of inverse micro-emulsions reaction system at different value of HLB.

No.	TX-100/n-Hexanol (Mass Ratio)	Ammonia/Cyclohexane (Mass Ratio)	HLB Value
a	9:0	1:10	13.50
b	3:1	1:10	11.63
c	3:2	1:10	10.50
d	1:1	1:10	9.75
e	3:4	1:10	9.21

**Table 3:** The component of inverse micro-emulsions with different molar ratios of water and surfactant (ω).

No.	TX-100/n-Hexanol (Mass Ratio)	Ammonia/Cyclohexane (Mass Ratio)	Mass of Distilled Water (g)	Molar Ratio of Water and Surfactant (ω)
a	3:2	1:15	0	9
b	3:2	1:15	0.75	12
c	3:2	1:15	1.75	16
d	3:2	1:15	2.75	20
e	3:2	1:15	5.75	32

**Table 4:** The component of inverse micro-emulsions with different concentrations of ammonium hydroxide.

No.	TX-100/n-Hexanol (Mass Ratio)	Ammonia/Cyclohexane (Mass Ratio)	Mass of Distilled Water (g)	Concentration of Ammonium Hydroxide (mol/L)	Molar Ratio of Water and Surfactant (ω)
a	3:2	1:45	4.25	0.15	20
b	3:2	1:22.5	3.5	0.3	20
c	3:2	1:15	2.75	0.44	20
d	3:2	1:11.25	2	0.58	20
e	3:2	1:10	1.625	0.65	20

**Table 5:** Properties of the prepared nano-silica with different silicon sources and methods.

Silicon Source	Method	Particle Size	The Specific Surface Area	References
Chlorosilane residual liquid	Inverse micro-emulsions	33.4 nm	850.5 m^2^/g	This study
Silicon tetrachloride	Low temperature vapor phase hydrolysis	141.7 nm	418 m^2^/g	[14]
Rice husk	Precipitation method	52.83 nm	618 m^2^/g	[15]

## Data Availability

Not applicable.
